# Association between Gut Microbiota and Biological Aging: A Two-Sample Mendelian Randomization Study

**DOI:** 10.3390/microorganisms12020370

**Published:** 2024-02-11

**Authors:** Chenglin Ye, Zhiqiang Li, Chun Ye, Li Yuan, Kailang Wu, Chengliang Zhu

**Affiliations:** 1 Department of Clinical Laboratory, Institute of Translational Medicine, Renmin Hospital of Wuhan University, Wuhan 430060, China; 2019283020200@whu.edu.cn (C.Y.);; 2 Department of General Surgery, Tongji Hospital, Tongji University School of Medicine, Shanghai 200065, China; 3 Department of Clinical Laboratory, Zhongnan Hospital of Wuhan University, Wuhan 430060, China; 4 State Key Laboratory of Virology, College of Life Sciences, Wuhan University, Wuhan 430072, China

**Keywords:** biological aging, aging, gut microbiota, mendelian randomization study, instrumental variables

## Abstract

Recent observational studies revealed an association between gut microbiota and aging, but whether gut microbiota are causally associated with the aging process remains unknown. We used a two-sample Mendelian randomization approach to investigate the causal association between gut microbiota and biological age acceleration using the largest available gut microbiota GWAS summary data from the MiBioGen consortium and GWAS data on biological age acceleration. We further conducted sensitivity analysis using MR-PRESSO, MR-Egger regression, Cochran Q test, and reverse MR analysis. *Streptococcus* (IVW, β = 0.16, *p* = 0.0001) was causally associated with Bioage acceleration. *Eubacterium* (*rectale group*) (IVW, β = 0.20, *p* = 0.0190), *Sellimonas* (IVW, β = 0.06, *p* = 0.019), and *Lachnospira* (IVW, β = −0.18, *p* = 0.01) were suggestive of causal associations with Bioage acceleration, with the latter being protective. *Actinomyces* (IVW, β = 0.26, *p* = 0.0083), *Butyricimonas* (IVW, β = 0.21, *p* = 0.0184), and *Lachnospiraceae* (*FCS020 group*) (IVW, β = 0.24, *p* = 0.0194) were suggestive of causal associations with Phenoage acceleration. This Mendelian randomization study found that *Streptococcus* was causally associated with Bioage acceleration. Further randomized controlled trials are needed to investigate its role in the aging process.

## 1. Introduction

With the continuous improvement in the medical field and quality of life, people’s life expectancy has generally been extended. However, the problem of aging has become increasingly serious [1]. According to the WHO’s predictions, the number of people over 60 years old in the global population will reach 2.1 billion by 2050 [2]. Aging is a major challenge that all countries in the world will face. Aging populations are severely affected by aging-associated diseases (AAD) and geriatric syndromes (GSs), which not only affect their quality of life but also create a significant burden on the social public health system [3]. Reversing the adverse effects of aging-associated diseases and geriatric syndromes is a difficult task, so the prevention and promotion of successful aging are particularly important. Traditionally, the process of aging is influenced by complex factors, including genetic and epigenetic factors as well as environmental factors [4]. Biological age refers to an assessment of an individual’s age based on various biological markers, health indicators, and physiological characteristics rather than simply relying on the passage of time as in chronological age. Biological aging, compared to chronological aging, offers several advantages in understanding the aging process [5]. Biological aging provides a more comprehensive and dynamic perspective on the aging process compared to chronological aging. This knowledge is crucial for developing interventions to promote healthy aging, prevent age-related diseases, and improve overall well-being in aging populations. In this article, we employed two metrics to measure biological aging. PhenoAge is computed based on chronological age and includes factors such as albumin, creatinine, C-reactive protein (CRP), alkaline phosphatase, glucose, lymphocyte percentage, mean corpuscular volume, red blood cell distribution width (RDW), and white blood cell count. On the other hand, BioAge, also contingent on chronological age, encompasses albumin, creatinine, CRP, and alkaline phosphatase (shared with PhenoAge). Additionally, Bioage incorporates glycated hemoglobin (HbA1c), systolic blood pressure, and total cholesterol. Both aging metrics, as established in previous studies [6,7], demonstrate robust predictive capabilities for aging-related outcomes.

Increasing evidence suggests that gut microbiota play an important role in the aging process [8]. The gut microbiome, the collection of microorganisms inhabiting the human gastrointestinal tract, emerged as a key player in regulating host physiology and health. The gut microbiota begin to colonize the body from birth and develop together with the individual, playing a role in different stages of an individual’s life. Accumulating evidence indicates that alterations in the gut microbiota composition and function, collectively referred to as dysbiosis, are associated with age-related diseases and may contribute to the aging process. Indeed, dysbiosis has been shown to affect systemic inflammation, immune function, and metabolism, all of which are hallmarks of aging [8,9,10,11]. Studies on some model animals suggested that gut dysbiosis may be a sign of aging [12,13,14]. Some studies show that gut microbiota diversity is higher in the high-longevity population [15]. A large-scale survey study of the elderly showed that the increase and decrease in the diversity of gut microbiota occur with actual age changes [16]. Some studies also show that supplementing certain gut microbiota can extend the lifespan of progeroid mice [17]. Investigating whether gut microbiota lead to accelerated aging or slow down aging, or whether other lifestyle and psychosocial factors play a role, is extremely challenging. Mendelian randomization (MR) provides powerful conditions for this purpose.

Observational studies cannot infer causal relationships between exposure and outcomes, and randomized controlled trial (RCT) studies often require a lot of research funding and costs and are constrained by experimental design limitations. Mendelian randomization uses genetic variation as an instrumental variable to infer causal relationships between exposures and outcomes from non-experimental data. It has been widely used as a novel research method. [18]. Using MR has identified causal relationships between gut microbiota and aging-related diseases such as cardiovascular diseases and neurodegenerative diseases [19,20]. MR studies also found causal relationships between gut microbiota and longevity [21,22]. However, no MR studies have yet demonstrated a causal relationship between gut microbiota and biological aging. In this study, MR was used to analyze the causal relationship between gut microbiota and biological aging in order to explore whether specific gut microbiota accelerate or decelerate the biological aging process and to provide new insights into promoting healthy aging through the modulation of gut microbiota.

## 2. Materials and Methods

### 2.1. Study Design and Ethics

**Figure 1:**
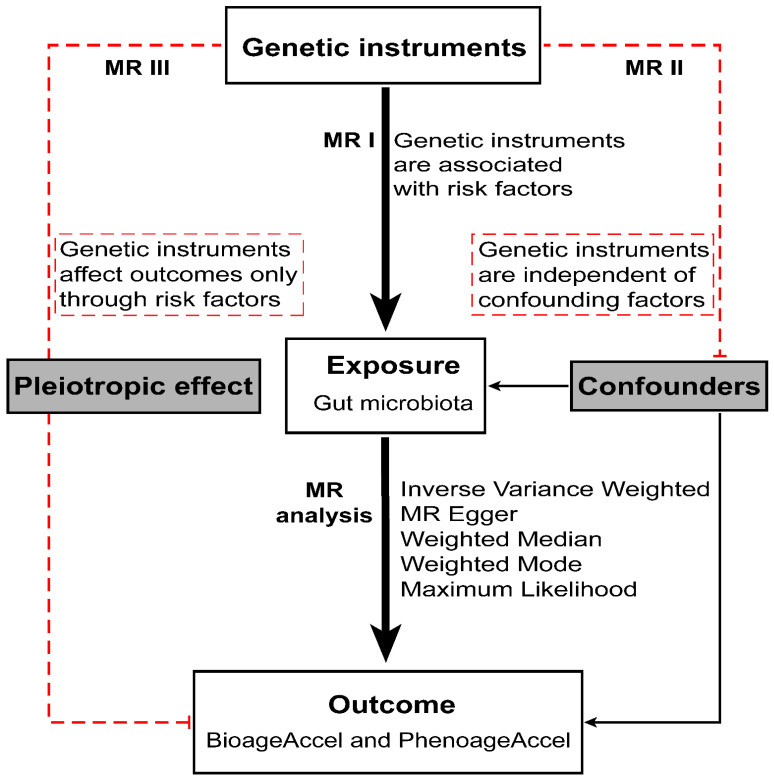
Overview of the design and methods used in this Mendelian randomization study. MR, Mendelian randomization; BioageAccel, Bioage Accelaration; PhenoageAccel, PhenoAge Accelaration; SNP, single-nucleotide polymorphism; se, standard error.

Our study design is illustrated in Figure 1. We used two-sample Mendelian randomization to investigate association between gut microbiota and biological aging. The study is based on publicly available data from MiBiogen consortium [23,24] and a study about biological aging carried out by Kuo et al. [5]. There is no sample overlap in the Genome-Wide Association Studies (GWAS) data between the exposure and the outcome. Each study is subject to the approval of the corresponding ethics committee.

### 2.2. Exposure Data Sources

GWAS summary data are based on the study of MiBiogen consortium, which provided genetic variants related to gut microbiota. This study coordinated genetic genotype data from 24 cohorts involving 18,340 participants, along with 16S ribosomal RNA (rRNA) gene sequencing profiles of fecal samples. The majority of participants had European ancestry (*n* = 13,266). Microbiota quantitative trait loci (mbQTL) mapping analysis was conducted to identify host genetic variants that were mapped to genetic loci associated with the abundance levels of bacterial taxa in the gut microbiota. Genus was the lowest taxonomic level analyzed, and the study identified 131 genera with a mean abundance greater than 1%. Our study analyzed data on 131 genera of European ancestry to investigate the association between gut microbiota and biological aging.

### 2.3. Outcome Data Sources

The GWAS summary data for biological age acceleration was obtained from Kuo et al.’s study. [5]. The study collected data on PhenoAge acceleration (PhenoAgeAccel) and Bioage acceleration (BioageAccel) from 107,460 and 98,446 individuals of European ancestry, respectively. PhenoAge and Bioage are two effective predictors of biological age. These two predictors were trained by Levine et al. using data from National Health and Nutrition Examination Survey (NHANES) III [6,7]. The calculation formulas and biological markers included in PhenoAge and Bioage can be found in Kuo et al.’s paper [5]. Kuo et al. validated PhenoAge and Bioage using data from the UK BioBank. Biological age acceleration is estimated by using the residuals of PhenoAge and Bioage after eliminating the effect of chronological age using linear regression models.

### 2.4. Statistical Analysis

We used a two-sample Mendelian randomization approach to analyze summary data. Following the basic principles of MR, we employed genetic variants as instrumental variables (IVs), which are required to fulfill three key assumptions: (1) the IVs used in the analysis must have an association with the gut microbiota, (2) the IVs must not be associated with any confounders, and (3) the IVs must affect the aging process (outcome variable) solely through the gut microbiota and not via any other pathway. We initially treated the gut microbiota as the exposure variable to assess its causal effects on the aging process. To obtain more single-nucleotide polymorphisms (SNPs) for subsequent sensitivity analyses, we referred to previous MR studies on the gut microbiota [25,26,27] and set a threshold of *p* < 1 × 10^−5^ to screen SNPs. In the reverse Mendelian randomization analysis, when BioageAccel and PhenoageAccel were used as exposure variables, we used a significance threshold of *p* < 5 × 10^−8^ to select SNPs. We evaluated the linkage disequilibrium (LD) of the selected SNPs using the 1000 Genomes Project European samples data and retained only the SNP with the lowest *p*-value, with an LD threshold of r^2^ < 0.001. To avoid weak instrument bias, we calculated the *F*-statistic. The formula for the *F*-statistic is as follows: $F=\frac{R^{2}\times (N-K-1)}{(1-R^{2})\times K}$, where *R*^2^ represents the proportion of variance in the exposure explained by genetic variation, *N* represents the sample size, and *K* represents the number of instrumental variables [28]. If the *F*-statistic is less than 10, it is considered a weak instrument. We used an online tool to calculate the power of the MR estimate [29,30].

We used multiple methods to infer causal associations, including the inverse variance weighted (IVW) method, MR-Egger, Weighted median, Weighted mode, and Maximum likelihood. The IVW method is the standard method for MR meta-analysis [28]. It does not require individual-level data and can directly calculate the causal effect size using summary data. If genetic variables are uncorrelated, the IVW estimate is equal to the estimate from the 2SLS method used for individual-level data [31]. The MR-Egger method relaxes the assumption of no pleiotropy among genetic variants in the IVW method. It assumes that the association between the instrument exposure and instrument outcome is independent, known as the instrument strength independent of direct effect (InSIDE) hypothesis [32]. This hypothesis is weaker than strict exclusion restriction criteria. However, both the IVW and MR-Egger regression methods theoretically assume that the genetic variant-exposure association is a measurement without error (no measurement error, NOME) [33]. The MR-Egger regression method violates the NOME assumption and results in greater bias than IVW estimates, particularly when affected by weak instrument bias. The Weighted median estimate takes into account the issue of large differences in estimation accuracy, requiring that only at least 50% of the weight is contributed by genetic variants [34]. When the InSIDE hypothesis is violated, the Weighted mode method is then shown to have higher statistical power for detecting a causal effect, less systematic error, and reduced type I error rates than the MR-Egger regression method [34]. The Maximum likelihood approach is similar to the IVW method, assuming no heterogeneity or horizontal pleiotropy. Assuming these assumptions are met, the obtained results will be unbiased, and the standard errors will be smaller compared to the IVW method [35]. In the sensitivity analyses, we used the MR pleiotropy residual sum and outlier (MR-PRESSO) method. The MR-PRESSO analysis identifies and endeavors to mitigate horizontal pleiotropy by eliminating noteworthy outliers. However, the MR-PRESSO outlier test mandates the validation of a minimum of 50% of the genetic variants as instruments and hinges on InSIDE assumptions [36]. We calculated the Cochran’s Q test to assess heterogeneity. This involves a weighted sum of the squared distances of the variant-specific estimates from the overall IVW estimate. A high value of the Q statistic indicates that the variant-specific ratio estimates differ more than expected due to chance alone. We used the MR-Egger regression to test for horizontal pleiotropy. To examine the causal relationship between gut microbiota and biological aging, we conducted a reverse MR analysis on the bacteria identified as causally linked to biological aging in the forward MR analysis. The methodologies used were in line with those applied in the forward MR analysis.

To avoid false positive results due to multiple testing, we employed the q-values to calculate False discovery rate and set the threshold for *q*-values at 0.05 [37]. A *p*-value < 0.05 but *q*-value > 0.05 was considered suggestive of a causal association. All statistical analyses were performed using R (version 4.2.2).

## 3. Results

Using summary-level data from GWAS meta-analyses of 131 genus-level gut microbiota as the exposure variables and GWAS meta-analyses of BioageAccel and PhenoageAccel as the outcome variables, we identified 3 to 21 SNPs with F-statistics ranging from 16.00 to 103.66, with no evidence of potential weak instrument bias (Tables S1–S4).

For Bioage acceleration, *Eubacterium* (*brachy group*) showed suggestive protective association in the IVW analysis (β = −0.06, standard error(se) = 0.03, *p* = 0.036, *q* = 0.77); in the Weighted median analysis, the association was suggestive (β = −0.10, se = 0.04, *p* = 0.0089, *q* = 0.97); and in the Maximum likelihood analysis, the β was −0.07 (se = 0.03, *p* = 0.0170, *q* = 0.29) (Table 1 and Table S3). For *Eubacterium* (*rectale group*), there was a suggestive association in the IVW analysis (β = 0.20, se = 0.08, *p* = 0.0190, *q* = 0.48) and a causal association in the Maximum likelihood analysis (β = 0.21, se = 0.06, *p* = 0.0008, *q* = 0.03). Since IVW was the main analysis, *Eubacterium* (*rectale group*) was considered to have a suggestive association. *Adlercreutzia* was found to have a causal association after the FDR correction in the IVW and Maximum likelihood analyses (IVW, β = 0.15, se = 0.04, *p* = 0.0004, *q* = 0.03; Maximum likelihood, β = 0.16, se = 0.04, *p* = 0.0005, *q* = 0.03). It showed a suggestive causal association in the Weighted median analysis (β = 0.14, se = 0.06, *p* = 0.015, *q* = 0.97) but had an opposite direction of effect in the MR-Egger analysis (β = −0.06, se = 0.19, *p* = 0.75, *q* = 0.86). *Bilophila* was found to have a suggestive causal association in the IVW and Maximum likelihood analyses (IVW, β = 0.09, se = 0.04, *p* = 0.042, *q* = 0.77; Maximum likelihood, β = 0.09, se = 0.04, *p* = 0.041, *q* = 0.51). *Lachnospira* was found to have a suggestive protective association (IVW, β = −0.18, se = 0.07, *p* = 0.01, *q* = 0.43; Weighted median, β = −0.18, se = 0.08, *p* = 0.029, *q* = 0.99; Maximum likelihood, β = −0.18, se = 0.07, *p* = 0.011, *q* = 0.23). *Sellimonas* was found to have suggestive association (IVW, β = 0.06, se = 0.03, *p* = 0.019, *q* = 0.48; Maximum likelihood, β = 0.06, se = 0.03, *p* = 0.011, *q* = 0.23). *Streptococcus* was causally associated with Bioage acceleration (IVW, β = 0.16, se = 0.04, *p* = 0.0001, *q* = 0.01; Maximum likelihood, β = 0.17, se = 0.04, *p* = 0.0001, *q* = 0.01).

When using PhenoAge acceleration as the outcome variable, *Actinomyces* was found to have suggestive associations (IVW, β = 0.26, se = 0.10, *p* = 0.0083, *q* = 0.54; Maximum likelihood, β = 0.27, se = 0.10, *p* = 0.0086, *q* = 0.25) (Table 2 and Table S4). *Butyricimonas* also had suggestive associations (IVW, β = 0.21, se = 0.09, *p* = 0.0184, *q* = 0.64; Maximum likelihood, β = 0.21, se = 0.09, *p* = 0.0189, *q* = 0.35). *Lachnospiraceae* (*FCS020 group*) had suggestive associations (IVW, β = 0.24, se = 0.10, *p* = 0.0194, *q* = 0.64; Maximum likelihood, β = 0.25, se = 0.10, *p* = 0.0104, *q* = 0.25). *Roseburia* had suggestive protective associations after FDR correction in the IVW analysis (β = −0.42, se = 0.14, *p* = 0.0034, *q* = 0.45) and remained protective even after FDR correction in the Maximum likelihood analysis (β = −0.42, se = 0.11, *p* = 0.0003, *q* = 0.03).

**Figure 2:**
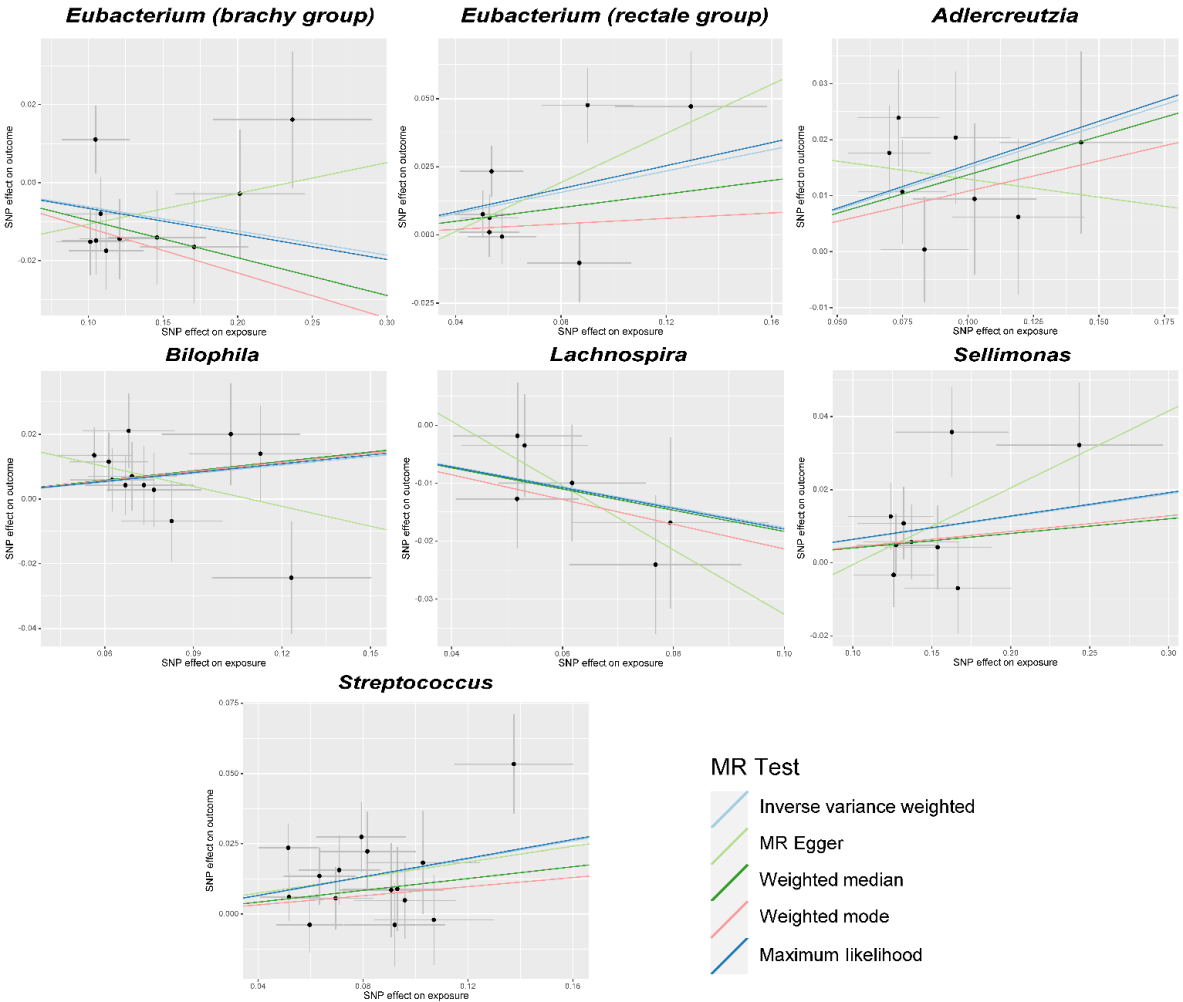
Scatter plots for the causal association between gut microbiota and Bioage acceleration. SNP, single nucleotide polymorphism; MR, Mendelian randomization.

**Figure 3:**
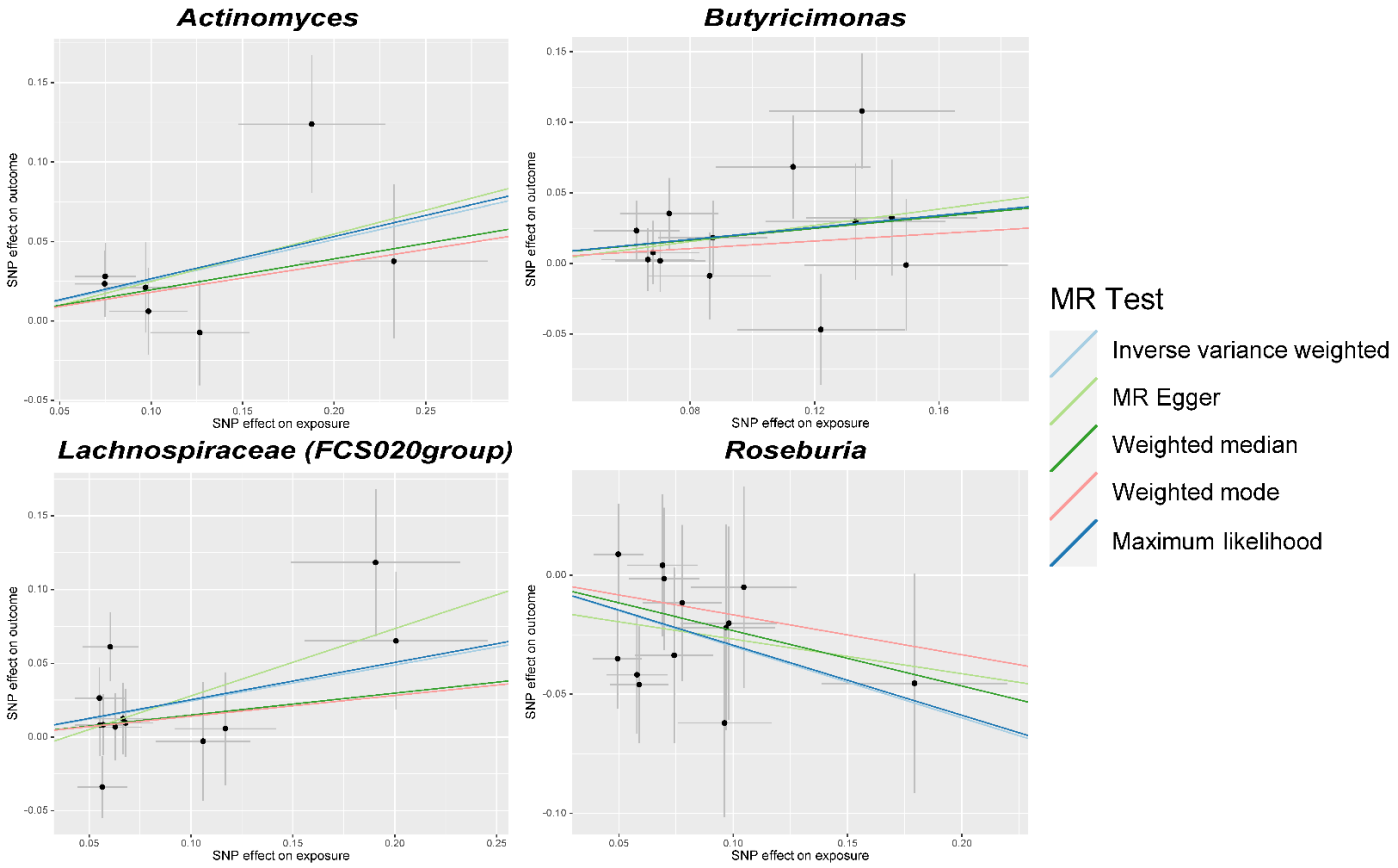
Scatter plots for the causal association between gut microbiota and PhenoAge acceleration. SNP, single nucleotide polymorphism; MR, Mendelian randomization.

All the gut microbiota mentioned above have causal or suggestive causal associations, except for *Roseburia* when PhenoAge acceleration was used as the outcome (Cochran’s IVW Q = 23.84, *p* = 0.033; Cochran’s MR Egger Q = 20.75, *p* = 0.054), showed no significant heterogeneity in their Cochran’s IVW Q and Cochran’s MR Egger Q values (Figure 2 and Figure 3; Tables S5–S10). MR-Egger regression intercept analysis did not reveal significant directional pleiotropy. Subsequent MR-PRESSO analysis found only one outlier SNP for *Eubacterium (rectale group)* in relation to Bioage acceleration (GlobalTest *p* = 0.0484), and no outliers were found for the others. After the MR-PRESSO analysis, the results for *Adlercreutzia*, *Bilophila, Lachnospira, Sellimonas*, and *Streptococcus* remained robust (*p* < 0.05) when the outcome was Bioage acceleration, and the results for *Actinomyces* and *Lachnospiraceae* (*FCS020 group*) remained robust (*p* < 0.05) when the outcome was PhenoAge acceleration. No obvious abnormal SNP was found in the subsequent leave-one-out analysis (Figures S1 and S2).

We further conducted a reverse Mendelian randomization analysis, and no significant causal associations were found when the exposure factor was Bioage acceleration, and the outcome factor was gut microbiota (Tables S11–S20). When the exposure factor was PhenoAge acceleration, only *Butyricimonas* showed a causal association in the MR Egger method (*p* = 0.0062), while IVW and other methods did not show significant causal associations. However, potential heterogeneity was detected in the IVs in the heterogeneity analysis (Cochran’s IVW Q = 68.94, *p* = 0.016), and potential horizontal pleiotropy was found in the MR-Egger regression intercept analysis (*p* = 0.015).

## 4. Discussion

Based on a review of the current literature, this study represents the first Mendelian randomization investigation into the potential causal relationship between gut microbiota and the aging process. Utilizing summary data from gut microbiota and aging-related GWAS, we conducted a two-sample Mendelian randomization analysis to explore the potential causal association between gut microbiota and accelerated aging. Our results show that the increase in *Streptococcus* abundance can accelerate aging, and *Eubacterium* (*rectale group*), *Sellimonas*, *Actinomyces*, *Butyricimonas*, *Lachnospiraceae* (*FCS020 group*), and *Lachnospira* have suggestive causal effects on aging acceleration and deceleration, respectively.

In this study, BioageAccel and PhenoageAccel were used to characterize the outcome variables of aging acceleration. Previous studies showed that Bioage and PhenoAge are reliable predictors of aging outcomes [6,7,38]. Both have been used to characterize biological age and aging acceleration in various studies [39,40,41,42]. Kuo et al. conducted a genome-wide association study on Bioage and PhenoAge and found that BioageAccel and PhenoageAccel were associated with cardiovascular metabolic risk and inflammation, respectively, both of which are closely related to the aging process [5]. Importantly, dysbiosis of the gut microbiota is a key factor in promoting cardiovascular and metabolic risk and systemic inflammation [43]. Therefore, we believe that BioageAccel and PhenoageAccel are powerful tools for quantifying the effect of gut microbiota on aging acceleration.

Based on existing research, there is evidence to suggest that dysbiosis of the gut microbiota is closely associated with the aging process. He et al. conducted a study to investigate the genetic correlation and causal relationship between gut microbiota and longevity using linkage disequilibrium score regression analysis and Mendelian randomization analysis. Their findings suggest a potential bidirectional causal relationship between gut microbiota and longevity [21]. Bárcena et al. found that both the progeria mouse model and clinical patients exhibited dysbiosis of gut microbiota. Moreover, the gut microbiota of centenarians exhibited a coexistence of healthy and pathogenic bacteria. Fecal microbiota transplantation (FMT) from wild-type donors to progeria recipients weakened the progeria phenotype, and the survival rate of progeria mice was also significantly improved [44]. Our study results indicate a causal relationship between *Streptococcus* and aging acceleration, which is of great significance for achieving anti-aging treatment by regulating gut microbiota and promoting healthy aging.

As a common gram-positive opportunistic pathogen, *Streptococcus* can exist in the nasopharynx and gut of healthy individuals. Under normal circumstances, it mostly does not have pathogenicity. However, when various internal and external factors cause disruption of the body’s microbiota, it can cause opportunistic infections and lead to purulent inflammation, scarlet fever, arthritis, acute glomerulonephritis, and other diseases. It was reported that dysbiosis of gut *Streptococcus* is associated with various diseases, such as atherosclerotic cardiovascular disease [45], hypertension [46], diabetes [47], obesity [48], colorectal cancer [49,50], lung cancer [51], gastric cancer [52], inflammatory bowel disease [50], mental disorders [53], multiple myeloma [54], systemic lupus erythematosus [55], and Parkinson’s disease [56]. Importantly, dysbiosis of gut *Streptococcus* is also closely related to the occurrence of aging-related diseases. Singh et al. employed 16S rDNA metagenomic sequencing analysis and reported a significant increase in the abundance of *Streptococcus* in the gut and oral microbiota of non-healthy aging individuals compared to healthy aging individuals [57]. Meanwhile, another study further showed that the gut microbiota of healthy longevity individuals had higher diversity, mainly dominated by *Bacteroides*, while the abundance of *Streptococcus* and other pathogenic bacteria in the gut microbiota of non-healthy longevity individuals was higher, leading to their abnormal biological metabolism and function in a non-healthy state [58].

It is important to note that opportunistic infections by intestinal *Streptococcus* can trigger an inflammatory response in the body, leading to the production of various inflammatory mediators within cells, such as IL-6, IL-8, and TNF-α [59,60,61,62]. These inflammatory mediators play a significant regulatory role in the aging process, accelerating the degradation and aging of cellular functions [63,64]. *Streptococcus* infections can also lead to increased intracellular oxidative stress, generating reactive oxygen species and free radicals, causing damage to cell structure and function [60,65]. Oxidative stress is one of the important triggers of cell aging [66], and *Streptococcus* may promote the aging process of cells by increasing oxidative stress levels. Furthermore, *Streptococcus* infections can induce changes in chromatin remodeling, leading to the relaxation and contraction of chromatin, affecting gene transcription and expression [67,68]. These changes in chromatin remodeling play a significant role in the cellular aging process [68,69].

*Eubacterium* (*rectale group*), *Sellimonas*, *Actinomyces*, *Butyricimonas*, and *Lachnospiraceae* (*FCS020 group*) were found to have suggestive accelerating effects on Bioage or PhenoAge in this study, while *Lachnospira* was found to have a suggestive protective effect on BioAge. Although *Eubacterium* (*rectale group*) is believed to produce butyrate and have anti-inflammatory effects [70], it has also been shown to promote inflammation and be associated with diabetic retinopathy and colon cancer [71,72]. *Sellimonas* has been reported to increase inflammatory diseases such as depression, ulcerative colitis, and ankylosing spondylitis [73,74]. *Lachnospira* and *Lachnospiraceae* (*FCS020 group*), although belonging to the same *Lachnospiraceae family*, have different effects on the body [75]. Studies found that the level of *Lachnospira* population in longevity village communities of the elderly is significantly higher than that in urbanized town communities [76], and *Lachnospira* levels are negatively correlated with asthma [77], depression and anxiety associated with ulcerative colitis [73], Parkinson’s disease [78], and psychiatric disorders [79]. Although *Butyricimonas* is a butyrate-producing bacterium, it can also cause bacteremia [80,81,82]. *Actinomyces*, as an opportunistic pathogen, is commonly colonized in the oral cavity, intestines, and urogenital tract [83]. Consistent with previous studies, the increase in the abundance of gut *Actinomyces* is associated with various inflammatory diseases, such as ulcerative colitis [84], Crohn’s disease [84,85], systemic lupus erythematosus [86], and COVID-19 [87,88]. A fundamental characteristic of aging is the presence of persistent low-grade inflammation, and the chronic inflammation caused by gut microbiota dysbiosis may be a potential mechanism for accelerating aging [63]. As people age, the abundance of beneficial microbes in the gut gradually decreases while that of pro-inflammatory microbes increases, which may lead to age-related diseases and premature death [89]. In contrast, although the gut microbiota also undergoes changes in long-lived individuals, its diversity and beneficial microbes are still preserved, thereby mitigating age-related inflammation and promoting healthy aging [21,58,90]. Importantly, disruptions in gut microbiota caused by various internal and external factors, such as improper diet and antibiotic use, can lead to a decrease in the ratio of beneficial to pro-inflammatory microbes. This, in turn, may promote inflammation and increase the risk of inflammation-related diseases, regardless of age [91]. Inflammation can lead to higher levels of reactive oxygen species (ROS), which can cause anaerobic *Firmicutes* in the gut to become inactive, exacerbating inflammation and promoting the occurrence of aging-related phenotypes [91,92].

This study has several strengths. Firstly, the study utilized a two-sample Mendelian randomization analysis method, which avoided bias from exposure and outcome summary levels and confounding factors on the results. Additionally, the use of multiple statistical methods in MR analysis minimized horizontal pleiotropy. The study utilized the largest published multi-cohort gut microbiota GWAS summary data, which minimized bias from differences in gut microbiota sequencing methods and ensured the representativeness of the results and the efficacy of the IV used in the MR analysis. Finally, the exposure and outcome data used in the study had no sample overlap, which reduced the occurrence of Type I errors caused by weak instrument bias [93].

This study also has several limitations. Mendelian randomization studies of the gut microbiota mainly focus on gut microbiota associated with genetic variation, while other gut microbiota weakly associated with individual genetic variation need further investigation using other research methods. Due to the limitations of the currently available gut microbiota GWAS summary data, this study included gut microbiota data at the genus level, limiting the investigation of the impact of species-level gut microbiota on aging. In order to include more instrumental variables for sensitivity analysis and horizontal pleiotropy testing, the SNPs used for analysis in this study were below the traditional GWAS significance threshold (*p* < 5 × 10^−8^). Aging is a complex process influenced by multiple factors, and although Bioage and PhenoAge can reflect aging to some extent, the overall characterization of aging still needs to be verified through larger sample GWAS studies and the inclusion of more dimensions of observational indicators. While biological age is considered a potentially significant indicator of the aging process, its predictive capacity in practical applications might vary across different populations, methodologies, and datasets. Aging processes vary widely among individuals due to genetic diversity, health status, and environmental exposures. This heterogeneity can complicate the identification of universal aging biomarkers or mechanisms. Since gut microbiota-related data in GWAS mainly come from European populations, further research and validation are needed to investigate the causal relationship between gut microbiota and accelerated aging in non-European populations.

The causal relationship between the gut microbiota and the complex trait of aging has always been a question worthy of exploration. However, conducting large-scale randomized controlled trials on the gut microbiota poses certain challenges. We have elucidated the causal relationship between specific microbial communities and aging through an alternative approach. It would be essential to delve deeper into the specific mechanisms underlying the causal relationship between gut microbiota and biological aging identified through MR analysis. Understanding the molecular pathways and interactions involved can provide insights into the precise ways in which gut microbiota influence biological aging processes. In terms of future outlook, continued research efforts in this area hold promise for uncovering novel strategies for promoting healthy aging and preventing age-related diseases. Integrating findings from MR analysis with other omics data, such as metagenomics, metabolomics, and transcriptomics, can provide a more comprehensive understanding of the complex interplay between gut microbiota and biological aging. Ultimately, insights gained from these investigations may lead to the development of personalized interventions to optimize gut microbial composition and promote healthy aging trajectories.

## 5. Conclusions

This two-sample Mendelian randomization study found that *Streptococcus* was causally associated with Bioage acceleration. Further randomized controlled trials are needed to investigate its role in the aging process. Other gut microbiota that showed suggestive causal relationships with the promotion or protection against aging also require further validation and exploration.

## Figures and Tables

**Table 1:** Mendelian randomization results of causal effects between gut microbiota and Bioage acceleration.

Exposure	No. of SNP	Method	*F*-Statistic	β	se	*p*	*q*-Value
*Eubacterium* (*brachy group*)	10	Inverse variance weighted	72.05	−0.06	0.03	0.0363	0.77
		MR−Egger		0.08	0.11	0.4880	0.86
		Weighted median		−0.10	0.04	0.0089	0.97
		Weighted mode		−0.12	0.06	0.1063	0.99
		Maximum likelihood		−0.07	0.03	0.0171	0.29
*Eubacterium* (*rectale group*)	8	Inverse variance weighted	18.28	0.20	0.08	0.0187	0.48
		MR-Egger		0.45	0.30	0.1849	0.86
		Weighted median		0.12	0.09	0.1706	0.99
		Weighted mode		0.05	0.14	0.7219	0.99
		Maximum likelihood		0.21	0.06	0.0008	0.03
*Adlercreutzia*	8	Inverse variance weighted	34.19	0.15	0.04	0.0004	0.03
		MR−Egger		−0.06	0.19	0.7474	0.86
		Weighted median		0.14	0.06	0.0147	0.97
		Weighted mode		0.11	0.09	0.2509	0.99
		Maximum likelihood		0.16	0.04	0.0005	0.03
*Bilophila*	12	Inverse variance weighted	22.41	0.09	0.04	0.0423	0.77
		MR−Egger		−0.21	0.19	0.3103	0.86
		Weighted median		0.10	0.06	0.0892	0.99
		Weighted mode		0.09	0.09	0.3117	0.99
		Maximum likelihood		0.09	0.04	0.0407	0.51
*Lachnospira*	6	Inverse variance weighted	18.13	−0.18	0.07	0.0101	0.43
		MR−Egger		−0.56	0.41	0.2478	0.86
		Weighted median		−0.18	0.08	0.0286	0.99
		Weighted mode		−0.21	0.12	0.1443	0.99
		Maximum likelihood		−0.18	0.07	0.0115	0.23
*Sellimonas*	9	Inverse variance weighted	103.66	0.06	0.03	0.0189	0.48
		MR−Egger		0.21	0.15	0.2022	0.86
		Weighted median		0.04	0.03	0.2444	0.99
		Weighted mode		0.04	0.05	0.4205	0.99
		Maximum likelihood		0.06	0.03	0.0111	0.23
*Streptococcus*	15	Inverse variance weighted	19.41	0.16	0.04	0.0001	0.01
		MR−Egger		0.14	0.16	0.3990	0.86
		Weighted median		0.11	0.06	0.0789	0.99
		Weighted mode		0.08	0.11	0.4728	0.99
		Maximum likelihood		0.17	0.04	0.0001	0.01

MR, Mendelian randomization; SNP, single nucleotide polymorphism; se, standard error.

**Table 2:** Mendelian randomization results of causal effects between gut microbiota and PhenoAge acceleration.

Exposure	No. of SNP	Method	*F*-Statistic	β	se	*p*	*q*-Value
*Actinomyces*	7	Inverse variance weighted	46.62	0.26	0.10	0.0083	0.54
		MR−Egger		0.30	0.27	0.3138	0.95
		Weighted median		0.20	0.13	0.1366	0.99
		Weighted mode		0.18	0.19	0.3781	0.98
		Maximum likelihood		0.27	0.10	0.0086	0.25
*Butyricimonas*	13	Inverse variance weighted	30.12	0.21	0.09	0.0184	0.64
		MR−Egger		0.29	0.30	0.3597	0.95
		Weighted median		0.21	0.12	0.0816	0.99
		Weighted mode		0.13	0.20	0.5130	0.98
		Maximum likelihood		0.21	0.09	0.0189	0.35
*Lachnospiraceae* (*FCS020 group*)	12	Inverse variance weighted	24.73	0.24	0.10	0.0194	0.64
		MR−Egger		0.46	0.26	0.1074	0.95
		Weighted median		0.15	0.14	0.2797	0.99
		Weighted mode		0.14	0.19	0.4635	0.98
		Maximum likelihood		0.25	0.10	0.0104	0.25
*Roseburia*	14	Inverse variance weighted	19.24	−0.42	0.14	0.0034	0.45
		MR−Egger		0.09	0.41	0.8333	1.00
		Weighted median		−0.24	0.15	0.1189	0.99
		Weighted mode		−0.17	0.21	0.4313	0.98
		Maximum likelihood		−0.42	0.11	0.0003	0.03

MR, Mendelian randomization; SNP, single nucleotide polymorphism; se, standard error.

## Data Availability

The GWAS data of gut microbiota can be obtained from the following website: MiBioGen, https://mibiogen.gcc.rug.nl/ (accessed on 20 March 2023). Biological aging acceleration GWAS data can be obtained from the following website: https://doi.org/10.6084/m9.figshare.12620291.v1 (accessed on 20 March 2023) and https://doi.org/10.6084/m9.figshare.12620366.v1 (accessed on 20 March 2023).

## Supplementary Materials

The following supporting information can be downloaded at: https://www.mdpi.com/article/10.3390/microorganisms12020370/s1. Additional file S1. (Supplementary Tables): Supplementary Table S1. Instrumental variables used in MR analysis of the association between gut microbiota and BioageAccel. Supplementary Table S2. Instrumental variables used in MR analysis of the association between gut microbiota and Phenoageaccel. Supplementary Table S3. Full result of MR estimates for the association between gut microbiota and BioageAccel. Supplementary Table S4. Full result of MR estimates for the association between gut microbiota and PhenoageAccel. Supplementary Table S5. The heterogeneity of gut microbiota instrumental variables (BioageAccel). Supplementary Table S6. The heterogeneity of gut microbiota instrumental variables (PhenoageAccel). Supplementary Table S7. Directional horizontal pleiotropy assessed by intercept term in MR Egger regression of the association between gut microbiota and BioageAccel. Supplementary Table S8. Directional horizontal pleiotropy assessed by intercept term in MR Egger regression of the association between gut microbiota and PhenoageAccel. Supplementary Table S9. MR-PRESSO analysis for the association between gut microbiota and BioageAccel. Supplementary Table S10. MR-PRESSO analysis for the association between gut microbiota and PhenoageAccel. Supplementary Table S11. Instrumental variables used in the MR analysis of the association between BioageAccel and gut microbiota. Supplementary Table S12. Instrumental variables used in the MR analysis of the association between PhenoageAccel and gut microbiota. Supplementary Table S13. Full result of MR estimates for the association between BioageAccel and gut microbiota. Supplementary Table S14. Full result of MR estimates for the association between PhenoageAccel and gut microbiota. Supplementary Table S15. The heterogeneity of BioageAccel instrumental variables. Supplementary Table S16. The heterogeneity of PhenoageAccel instrumental variables. Supplementary Table S17. Directional horizontal pleiotropy assessed by intercept term in MR Egger regression of the association between BioageAccel and gut microbiota. Supplementary Table S18. Directional horizontal pleiotropy assessed by intercept term in MR Egger regression of the association between PhenoageAccel and gut microbiota. Supplementary Table S19. MR-PRESSO analysis for the association between BioageAccel and gut microbiota. Supplementary Table S20. MR-PRESSO analysis for the association between PhenoageAccel and gut microbiota. Supplementary Table S21. Details of studies and datasets used for analyses. Additional file S2. (Supplementary Figures): Supplementary Figure S1. Leave one out analysis between gut microbiota and BioAge acceleration. Supplementary Figure S2. Leave one out analysis between gut microbiota and PhenoAge acceleration.

## Abbreviations

AAD: aging-associated diseases; BioAgeAccel: BioAge Accelaration; FDR: False discovery rate; FMT: Fecal microbiota transplantation; GSs: geriatric syndromes; GWAS: Genome-Wide Association Studies; InSIDE: instrument strength independent of direct effect; IVs: instrumental variables; IVW: inverse variance weighted; LD: linkage disequilibrium; mbQTL: Microbiota quantitative trait loci; ML: Maximum likelihood; MR: Mendelian randomization; MR-PRESSO: MR pleiotropy residual sum and outlier; NHANES: National Health and Nutrition Examination Survey; NOME: no measurement error; PhenoAgeAccel: PhenoAge Accelaration; RCT: randomized controlled trial; ROS: reactive oxygen species; SE, standard error; SNPs: single nucleotide polymorphisms.
