# Supplementary material for: Association between Gut Microbiota and Biological Aging: A Two-Sample Mendelian Randomization Study
Source: Microorganisms. 2024 Feb 11;12(2):370. doi: 10.3390/microorganisms12020370 (PMC10891714; doi:10.3390/microorganisms12020370)
Supplement: Supplementary file 1 [file microorganisms-12-00370-s001.zip › Figure_S1.pdf]

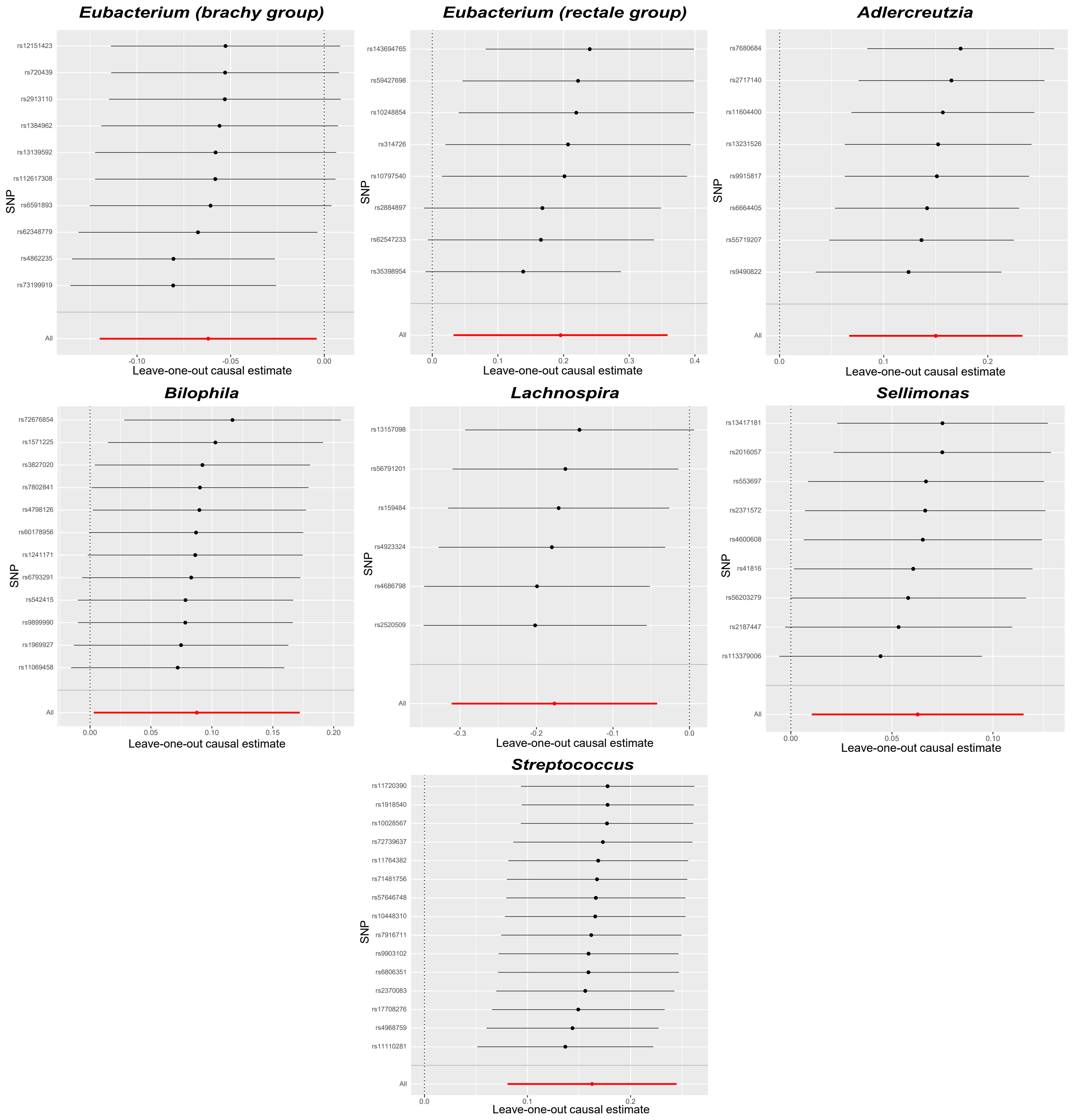
Eubacterium (brachy group)
rs12151423
rs720439
rs2913110
rs1384962
rs13139592
rs112617308
rs6591893
rs62348779
rs4862235
rs73199919
All
SNP
-0.10
-0.05
0.00
Leave-one-out causal estimate
Eubacterium (rectale group)
rs143694765
rs59427698
rs10248854
rs314726
rs10797540
rs2884897
rs62547233
rs35398954
All
SNP
0.0
0.1
0.2
0.3
0.4
Leave-one-out causal estimate
Adlercreutzia
rs7680684
rs2717140
rs11604400
rs13231526
rs9915817
rs6664405
rs55719207
rs9490822
All
SNP
0.0
0.1
0.2
Leave-one-out causal estimate
Bilophila
rs72676854
rs1571225
rs3827020
rs7802841
rs4798126
rs60178956
rs1241171
rs6793291
rs542415
rs9899990
rs1969927
rs11069458
All
SNP
0.00
0.05
0.10
0.15
0.20
Leave-one-out causal estimate
Lachnospira
rs13157098
rs56791201
rs159484
rs4923324
rs4686798
rs2520509
All
SNP
-0.3
-0.2
-0.1
0.0
Leave-one-out causal estimate
Sellimonas
rs13417181
rs2016057
rs553697
rs2371572
rs4600608
rs41816
rs56203279
rs2187447
rs113379006
All
SNP
0.00
0.05
0.10
Leave-one-out causal estimate
Streptococcus
rs11720390
rs1918540
rs10028567
rs72739637
rs11764382
rs71481756
rs57646748
rs10448310
rs7916711
rs9903102
rs6806351
rs2370083
rs17708276
rs4968759
rs11110281
All
SNP
0.0
0.1
0.2
Leave-one-out causal estimate
