# Supplementary figures and images for: Association between Gut Microbiota and Biological Aging: A Two-Sample Mendelian Randomization Study
Source: Microorganisms. 2024 Feb 11;12(2):370. doi: 10.3390/microorganisms12020370 (PMC10891714; doi:10.3390/microorganisms12020370)

### *Actinomyces*

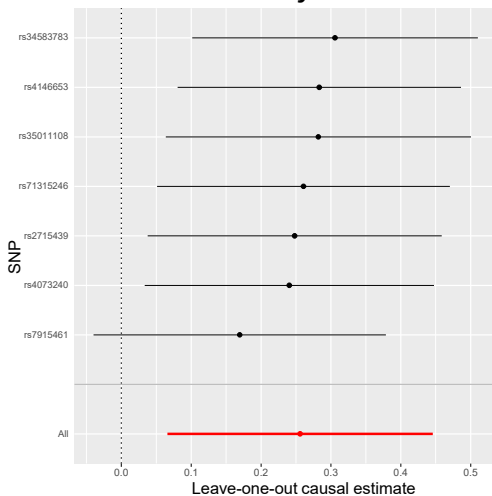

### *Butyrivimonas*

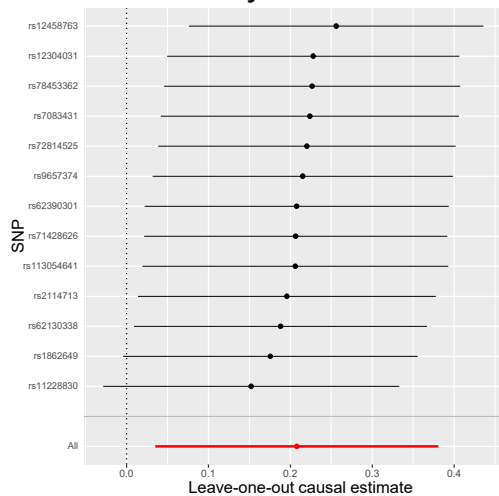

### *Lachnospiraceae (FCS020group)*

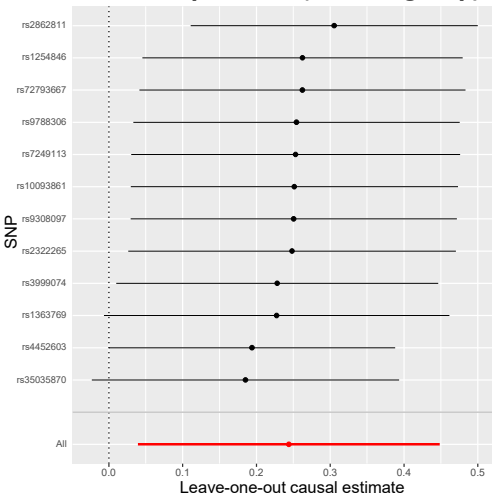

### *Roseburia*

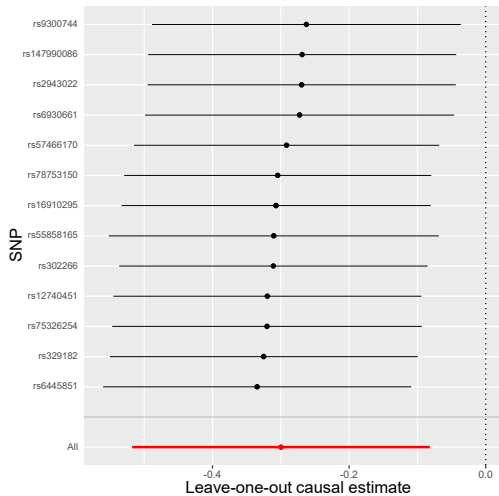

Supplement: Supplementary file 1 [file microorganisms-12-00370-s001.zip › Figure_S2.pdf]
